# Motivation, Social Emotion, and the Acceptance of Artificial Intelligence Virtual Assistants—Trust-Based Mediating Effects

**DOI:** 10.3389/fpsyg.2021.728495

**Published:** 2021-08-13

**Authors:** Shiying Zhang, Zixuan Meng, Beibei Chen, Xiu Yang, Xinran Zhao

**Affiliations:** ^1^Business and Economic Research Institute, Harbin University of Commerce, Harbin, China; ^2^School of Business, Dalian University of Technology, Dalian, China; ^3^Accounting and Auditing College, Guangxi University of Finance and Economics, Nanning, China

**Keywords:** AI virtual assistant, motivation, social emotion, trust, acceptance, mediating effects, inverted U relationship

## Abstract

The complexity of the emotional presentation of users to Artificial Intelligence (AI) virtual assistants is mainly manifested in user motivation and social emotion, but the current research lacks an effective conversion path from emotion to acceptance. This paper innovatively cuts from the perspective of trust, establishes an AI virtual assistant acceptance model, conducts an empirical study based on the survey data from 240 questionnaires, and uses multilevel regression analysis and the bootstrap method to analyze the data. The results showed that functionality and social emotions had a significant effect on trust, where perceived humanity showed an inverted U relationship on trust, and trust mediated the relationship between both functionality and social emotions and acceptance. The findings explain the emotional complexity of users toward AI virtual assistants and extend the transformation path of technology acceptance from the trust perspective, which has implications for the development and design of AI applications.

## Introduction

With the advancement of AI technology, there are increasing numbers of Artificial Intelligence (AI) applications, such as service robots, chatbots, and AI virtual assistants (Gummerus et al., 2019). Regarding AI virtual assistants, which can offer convenience and more efficient services to users (van Doorn et al., 2017; Fernandes and Oliveira, 2021), people's interest and frequency of use is gradually increasing. Since technology acceptance is a key variable reflecting whether AI virtual assistants are accepted by users (Fernandes and Oliveira, 2021), it is important for product developers and corporate investors to explore the drivers of AI virtual assistant acceptance and their mechanisms of action.

However, current research on the acceptance of AI virtual assistants still suffers from the following three deficiencies. First, the current studies focus more on the impact of functionality and social emotions (Wirtz et al., 2018) than on the acceptance of AI virtual assistants (AVA), which helps reveal consumers' intention to use AI virtual assistants. However, the dual satisfaction of technical and social needs does not induce users to trust AI virtual assistants, which leads to low loyalty. In this case, it will be difficult for human users to collaborate with AI virtual assistants, thus limiting their application in society and making it difficult for AI virtual assistants to be truly accepted by human users.

In fact, trust can reduce human users' negative emotions about and affect their tendency to accept new technologies (Sparks and Browning, 2011). Consequently, the applicability of trust in the field of AI still needs further verification. Second, some scholars have tried to explore the potential mechanisms of users' trust in AI virtual assistants from the perspective of trust (Hassanein and Head, 2007; Glikson and Woolley, 2020); however, the relationship between trust and functionality and social emotions and whether the existing drivers have an impact on trust remain to be further explored. Finally, there is still a lack of effective transformation paths between AVA and their drivers, and it is unclear whether trust can carry the transformation between the two. This research scenario is not conducive to expanding the potential paths and intrinsic functions of AVA from a trust perspective.

This paper describes the following research findings. First, trust can reduce users' rejection of new things, thus promoting users' acceptance of AI virtual assistants at the psychological level, which is a direct driver of AI virtual assistant acceptance. Second, trust building depends on the user's motivation and perception of using the AI virtual assistant, that is, it is affected by functionality and social emotion. In this process, a positive experience contributes to user trust; for example, users are more concerned with whether something is useful or convenient. Nevertheless, users' perceptions of AI virtual assistants hardly have a coherent effect on trust, which includes the positive effects of perceived social presence and perceived social interaction as well as the inverted U-shaped effects of perceived humanity. In other words, the satisfaction of users' social needs by AI virtual assistants can effectively increase users' trust in them, but as perceived humanity tends to contribute to the trust transition effect, AI virtual assistants should be designed to maintain a moderate level of perceived humanity so that users can trust their services better. Finally, this paper reveals the transformation path between functionality, social emotion and AVA, namely, the mediation effect of trust is examined. In this procedure, trust presents two different effect mechanisms, which are partially mediated between functionality and acceptance and fully mediated between social emotion and acceptance, with two different degrees of mediation effects also indicating the effectiveness of relying on trust as a transformation path.

## Research Framework and Hypothesis Development

**Figure 1:**
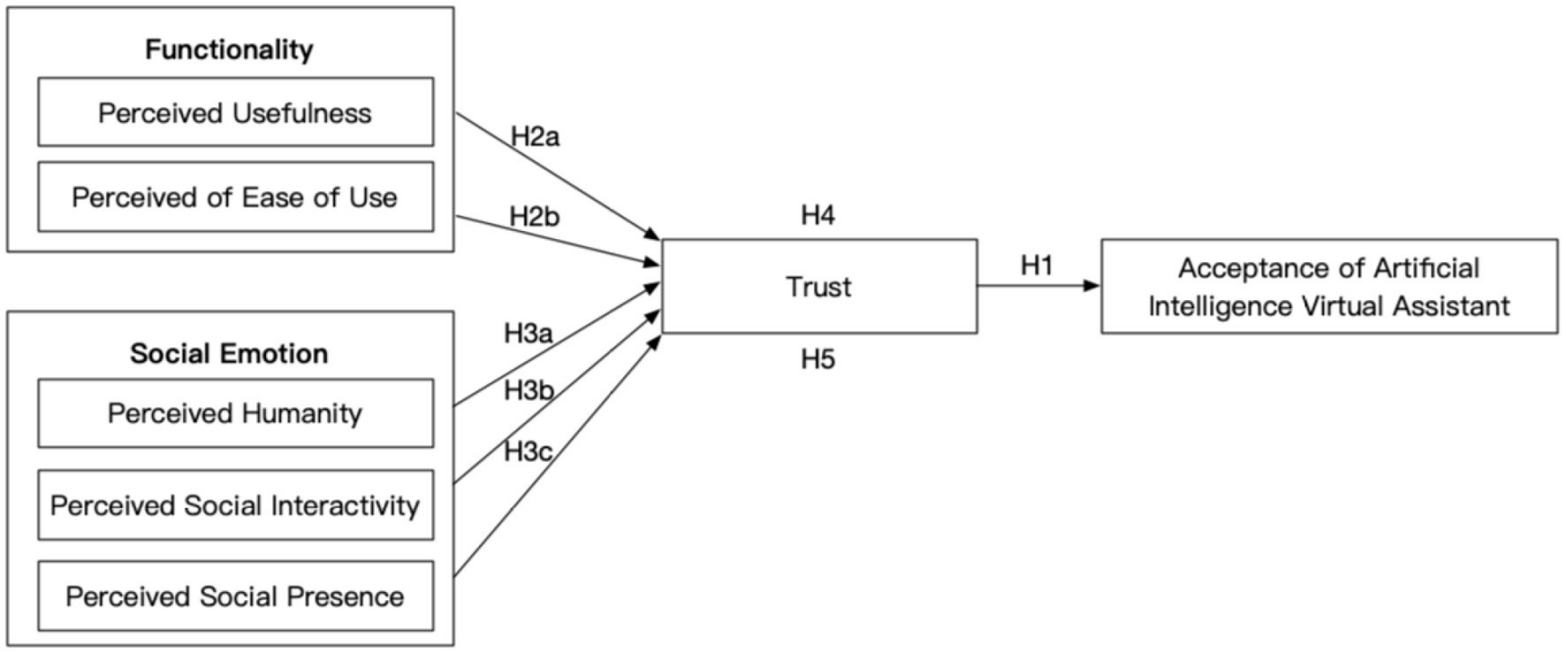
Artificial intelligence virtual assistant acceptance model.

Referring to the technology acceptance model and service robot acceptance model, this paper contains three levels of research on AVA: first, it investigates the relationship between trust and acceptance; second, it investigates the relationship between trust and functionality and social emotion; third, it investigates the mediating effect of trust between both functionality and social emotion and acceptance. Based on the above theoretical models, this study's theoretical model and hypotheses are shown in Figure 1.

Trust is defined as the user's confidence that the AI virtual assistant can reliably deliver a service (Wirtz et al., 2018). The services of AI virtual assistants are based on artificial intelligence algorithms, but due to the inherent black box problem of artificial intelligence technology (Asatiani et al., 2020), users will not fully trust the information or services provided by artificial intelligence virtual assistants (Kaplan and Haenlein, 2019). The existing research shows that only meeting the technical and social needs of users does not truly increase their loyalty to AI virtual assistants (Hassanein and Head, 2007). Trust will prompt users to subjectively reduce their negative emotional perception of AI virtual assistants, which contributes to their reduced complexity and vulnerability and plays a key role in improving the acceptance of AI virtual assistants (Shin, 2021). Therefore, this paper introduces trust variables to explore the mechanism underlying their effect on acceptance and makes the following hypothesis:

**H1**: Users' behavior of trusting AI virtual assistants is positively correlated with the AVA.

Perceived usefulness is the degree to which an individual perceives that a technology improves their performance and is an important factor in determining user acceptance, adoption, and usage (Kulviwat et al., 2007; Jan and Contreras, 2011). Perceived ease of use is the degree to which an individual's perception that using a technology requires minimal physical and mental effort, which is an important driver of technology acceptance and adoption (Kulviwat et al., 2007). Wirtz et al. (2018) put perceived usefulness and perceived of ease of use at the core of the service robot acceptance model. McLean and Osei-Frimpong (2019) found that utility benefits (namely, perceived usefulness and perceived of ease of use) have a positive impact on users' use of AI virtual assistants. The results show that perceived usefulness and perceived of ease of use are important antecedents that influence the formation of consumer trust. Venkatesh and Bala (2008), on the other hand, found that perceived usefulness and perceived of ease of use were significant predictors of behavioral intention. Perceived usefulness and perceived of ease of use have a positive effect on individuals' acceptance of the technology, and the higher the indicators of P perceived usefulness and perceived of ease of use, the more positive are users' attitudes toward the AI virtual assistant, which leads to trust in the AI virtual assistant. Glikson and Woolley (2020) argued that trust formation also depends upon machine competence (i.e., the extent to which it does its job properly). Therefore, this paper makes the following hypotheses.

**H2a**: Perceived usefulness is positively correlated with users' behavior of trusting AI virtual assistants.
**H2b**: Perceived ease of use is positively correlated with users' behavior of trusting AI virtual assistants.

Perceived humanity, also known as anthropomorphism, refers to whether the user perceives the AI virtual assistant as human during interaction with it. Perceived humanity is an important determinant of customer use of AI virtual assistants (van Doorn et al., 2017). Scholars hold different views on, with research showing that users tend to use anthropomorphized AI assistants (Epley et al., 2008). Drawing on the “Uncanny Valley” effect, a highly anthropomorphic AI virtual assistant will make users more inclined to measure human-computer interaction by the rules of human interaction and form higher expectations. When the AI virtual assistant makes a low-level mistake, the inconsistency between the high form of anthropomorphism and the mistake behavior defies the user's expectation and creates a sense of disgust. Once the AI virtual assistant is anthropomorphized, the user will experience a sense of connection with it (van Pinxteren et al., 2019), but because the AI virtual assistant is not human, it will create a sense of unnaturalness and may even cause the user's interaction with the AI virtual assistant to be completely interrupted (Tinwell et al., 2011; van Doorn et al., 2017). Users who are more sensitive to perceived humanity believe that AI virtual assistants with human-like characteristics threaten human specificity and self-identity (Gursoy et al., 2019). In addition, humans must interact with AI virtual assistants and learn how to interact with AI virtual assistants, thus increasing the burden on consumers to use AI devices (Kim and McGill, 2018). According to existing research, moderate perception of humanity will enhance the trust between human users and AI virtual assistants, while excessive perception of humanity will make human users feel threatened or even fearful, which may even cause human users to interrupt their interactions. Based on this, the paper makes the following assumptions:

**H3a**: Perceived humanity has an inverted U-shaped relationship with user trust in artificial intelligence virtual assistants.

Interaction means that people connect with information through interaction, in which they communicate and exchange emotions, energy, resources and other contents while generating judgments and reactions to the activities and words of others. From the perspective of information dissemination, interaction is based on the relationship between people and computers, using new technologies to enhance the interaction between users and computers (Shin, 2020). Current research suggests that interaction promotes both emotional and behavioral loyalty to technology (Wirtz et al., 2018; Sundar, 2020) and that enhanced interactions can increase user satisfaction with a website (Song and Zinkhan, 2008; Jiang et al., 2019). Hence, the interactivity of AI virtual assistants can engage users with positive effects. Perceived social interactivity can be defined as the perception that the AI virtual assistant displays appropriate actions and “emotions” according to societal norms (Wirtz et al., 2018). If an AI virtual assistant interacts in a social manner, demonstrates its social capabilities, and provides favorable service to the user, its social appeal increases (McLean and Osei-Frimpong, 2019), thus promoting trust in it (Chattaraman et al., 2019). Therefore, the following assumptions are made in this paper:

**H3b**: Perceived social interactivity is positively correlated with user trust in artificial intelligence virtual assistants.

Perceived social presence refers to the degree to which the user perceives the AI virtual assistant as a social entity. Drawing on social presence theory, perceived social presence is an inherent quality of AI virtual assistants. Perceived social presence means that the user has a perception of human interaction with the AI virtual assistant that is personal, social, warm and sensitive. If an AI virtual assistant conveys a sense of interpersonal and social connection to the user, the user will have a positive experience with it (Holzwarth et al., 2006; Wirtz et al., 2018) and perceive the AI virtual assistant as a real social entity. In cases where AI virtual assistants demonstrate a higher perceived social presence, users will build stronger trust in them (Wang and Emurian, 2005). Moreover, AI virtual assistants have real-time communication, voice, politeness, and other language-based communication skills, which can meet the social needs of human users and generate positive emotions, creating a harmonious social atmosphere, thus prompting human users to develop trust in AI virtual assistants (Fernandes and Oliveira, 2021). Therefore, the following assumptions are made in this paper.

**H3c**: Perceived social presence is positively correlated with user trust in AI virtual assistants.

Functionality refers to the degree of usefulness and convenience of the AI virtual assistant. Social emotion refers to the social experience of human users during their interaction with the AI virtual assistant. However, there is no effective transformation path between the two dimensions and acceptance; therefore, this paper introduces the trust variable to explore its mechanism of action between usage motivation and perception and acceptance. According to the brand effect, AI virtual assistants that can provide favorable services and information tend to bring users a sense of cozy experience, forming a positive cycle, which means that a positive product usage experience drives users to trust AI virtual assistants more, thus increasing their loyalty and acceptance. In environments lacking social emotion, users tend to hide information and reduce their trust behavior. Therefore, users will trust AI virtual assistants more in contexts where social emotions are stronger (Glikson and Woolley, 2020); that is, social emotions are necessary for trust development (Hassanein and Head, 2007). Research shows that trust is influenced by both rational (i.e., functionality) and emotional (i.e., social emotion) dimensions, and trust mediates between the rational and emotional dimensions and user acceptance (Palmatier et al., 2006; Glikson and Woolley, 2020). If the AI virtual assistant can bring more trust to the user, it will reduce the user's suspicion of it, and it will improve the AVA. Therefore, this paper explores the mediating role of trust between functionality and social emotion and acceptance and proposes the following hypotheses:

**H4**: User trust behavior has a mediating role between functionality and acceptance.
**H5**: User trust behavior has a mediating role between social emotion and acceptance.

## Data and Research Methodology

### Scale Design and Data Sources

At present, AI virtual assistants are widely used in daily life, and people of all ages and industries are able to access the use of AI virtual assistants and can grasp the scenario of this study more accurately. Therefore, this paper selects the public as the investigation target. This paper uses Wenjuanxing to generate online questionnaires and sends out questionnaire links and QR codes through WeChat groups, friend circles, QQ groups, online forums, and so on to invite the public to visit the questionnaire. This paper collects sample data with the help of the Questionnaire Star platform and sends questionnaires by QQ, WeChat, friends circles, among other routes. A total of 240 valid questionnaires were obtained. The descriptive statistics of the sample are shown in Table 1.

**Table 1:** Results of descriptive statistics of the sample (*N* = 240).

**Content**	**Category**	**Sample size**	**Proportion (%)**
Gender	Male	101	42.08
	Female	139	57.92
Age	20<	36	15
	21–25	111	46.25
	26–30	32	13.33
	31–35	27	11.25
	36–40	13	5.42
	>41	21	8.75
Education	Undergraduate	152	63.33
	Master	37	15.42
	Ph.D.	8	3.33
	Other	43	17.92
Marital status	Unmarried	153	63.75
	Married with children	70	29.17
	Married with no children	17	7.08

### Variable Measurement

To ensure the reliability and validity of the measurement scales, mature measurement scale items were selected for this study and appropriately adapted to the study scenario. In particular, functionality is based on the technology acceptance model and incorporates the findings of Venkatesh and Davis (2000) and others to classify functionality into two dimensions, perceived usefulness and perceived of ease of use, each of which contains four question items. Social emotion is based on the service robot acceptance model, which is divided into three dimensions: perceived humanity, perceived social interactivity, and perceived social presence. Combined with the study of Fernandes and Oliveira (2021), perceived humanity contains four question items, perceived social interactivity contains two question items, and perceived social presence contains three question items. Trust is based on the service robot acceptance model, containing one dimension of trust and four question items based on Shin (2021). The AVA contains three question items according to Fernandes and Oliveira (2021). The question items in this paper are all scored on a 5-point Likert scale, with 1–5 indicating very poor to fully conforming.

### Reliability and Validity Tests

The descriptive statistics and correlation coefficients are shown in Table 2. The results show that perceived usefulness is positively related to trust (*r* = 0.510, *p* < 0.01), perceived of ease of use had a positive correlation with Trust (*r* = 0.464, *p* < 0.01), perceived social interactivity displayed a positive correlation with trust (*r* = 0.547, *p* < 0.01), and perceived social presence is found to be positively correlated with trust (*r* = 0.537, *p* < 0.01). These results preliminarily supported hypotheses 2a, 2b, 3b, and 3c.

**Table 2:** Descriptive statistics results with correlation coefficients.

**Variables**	**Average**	**Standard deviation**	**1**	**2**	**3**	**4**	**5**	**6**	**7**
1 Perceived usefulness	3.579	0.835	1						
2 Perceived of ease of use	3.561	0.719	0.722^**^	1					
3 Perceived humanity	2.79	1.062	0.325^**^	0.162^*^	1				
4 Perceived social interactivity	3.333	0.911	0.679^**^	0.594^**^	0.463^**^	1			
5 Perceived social presence	3.189	1.026	0.555^**^	0.469^**^	0.551^**^	0.714^**^	1		
6 Trust	3.526	0.708	0.510^**^	0.464^**^	0.320^**^	0.547^**^	0.537^**^	1	
7 AVA	3.782	0.727	0.634^**^	0.528^**^	0.141^*^	0.506^**^	0.432^**^	0.467^**^	1

* *p < 0.05*

** *p < 0.01*.

As shown in Table 3, the Cronbach α of the seven variables are >0.7, indicating that factor analysis was appropriate.

**Table 3:** Reliability test results.

**Variables**	**Items**	**Cronbach α**
Perceived usefulness	4	0.794
Perceived of ease of use	4	0.906
Perceived humanity	4	0.924
Perceived social interactivity	2	0.733
Perceived social presence	3	0.898
Trust	4	0.818
AVA	3	0.871

Confirmatory factor analysis was conducted using SPSSAU to directly test the validity through confirmatory factor analysis of mature scales (as shown in Table 4). The results showed that standard load factors were all in acceptable range (>0.400), which indicates a strong correlation between the latent variables and the analytic term measures. And the results show that the AVE is >0.5 and the CR value is >0.7, which means the aggregation validity is high.

**Table 4:** Scale items and validity tests.

**Factor (latent variable)**	**Measurement items (significant variables)**	**Standard load factor**
		
Perceived usefulness (CR = 0.906, AVE = 0.708)		
	1. I find that using an AI virtual assistant will improve my daily work performance	0.817
	2. I find that using an AI virtual assistant will help me in my daily work	0.866
	3. I find that using an AI virtual assistant will improve my daily work productivity	0.860
	4. I find that using an AI virtual assistant will be useful for my daily work	0.821
Perceived of ease of use (CR = 0.809, AVE = 0.523)		
	1. I think it will be easy to use the AI virtual assistant	0.778
	2. I find that the interaction with the AI virtual assistant is clear and easy to understand	0.841
	3. I find that the AI virtual assistant is difficult to use	0.533
	4. I find that it is easy to get the AI virtual assistant to do what I want it to do	0.692
Perceived humanity (CR = 0.926, AVE = 0.759)		
	1. I think the AI virtual assistant has a mind of its own	0.874
	2. I think the AI virtual assistant has consciousness	0.933
	3. I think the AI virtual assistant has its own free will	0.900
	4. I think the AI virtual assistant can experience emotions	0.773
Perceived social interactivity (CR = 0.743, AVE = 0.595)		
	1. I think the AI virtual assistant is easy to get along with	0.705
	2. I think the AI virtual assistant can understand me	0.824
Perceived social presence (CR = 0.900, AVE = 0.751)		
	1.There is a sense of interacting with a human being when interacting with an AI virtual assistant	0.843
	2. There is a sense of social interaction with the AI virtual assistant	0.892
	3. There is a sense of humanity in interacting with the AI virtual assistant	0.864
Trust (CR = 0.819, AVE = 0.534)		
	1. In my experience, the AI virtual assistants are honest	0.692
	2. In my experience, the AI virtual assistant cares about the user	0.772
	3. In my experience, the AI virtual assistant provides favorable service	0.688
	4. In my experience, the AI virtual assistant is trustworthy	0.752
AVA (CR = 0.872, AVE = 0.694)		
	1. I will try to use an AI virtual assistant in the future	0.807
	2. I plan to use the AI virtual assistant in the future	0.848
	3. I intend to use the AI virtual assistant in the future	0.841

The results of KMO and Bartlett's test are shown in Table 5. The KMO value is more than 0.9 and *p*-value is < 0.05, indicating that the validity of the study data was feasible.

**Table 5:** KMO and Bartlett's test.

**KMO value**		**0.914**
Bartlett Sphericity test	Approximate cardinality	4155.013
	df	276
	*p*-value	0

## Empirical Testing and Analysis

### Selection of Research Method

The purpose of this study is to investigate the factors influencing acceptance and their mechanisms of action. Based on the existing literature, this paper identifies a study of acceptability consisting of four dimensions: functionality, social emotion, trust, and AVA. This paper uses multilevel regression analysis to analyze the correlations among the variables. The core of multilevel regression analysis is regression analysis; the difference is that hierarchical regression can be divided into multiple layers, where each layer adds more polynomials on top of the previous layer. This approach can solve the problem of whether more polynomials have explanatory power for the model. In addition, this paper used the product coefficient test for mediating effects; specifically, the bootstrap sampling method was used for testing. The basic idea of bootstrap sampling is to construct an estimated confidence interval with the help of multiple sampling with partial sample release when the full sample is unknown. This method has relatively high test efficacy and does not impose restrictions on the mediating sampling distribution.

### Correlation Test

In this paper, a multilevel regression analysis was used to test the hypotheses using SPSSAU software.

As shown in Table 6, this stratified regression analysis involved a total of three models. There are three models involved in this hierarchical regression analysis. The independent variables in model 1 are control variables (Gender, Age, Education and Marital Status), model 2 adds perceived of ease of use and perceived usefulness to model 1, and model 3 adds perceived humanity, perceived humanity squared, perceived social interactivity, and perceived social presence to model 2.

**Table 6:** Results of multilevel regression tests (Explanatory variable: Trust).

**Category**	**Variable**	**Model 1**	**Model 2**	**Model 3**
Control variables	Gender	−0.010 (−0.110)	−0.015 (−0.189)	0.049 (0.734)
	Age	0.052 (1.368)	0.051 (1.575)	0.039 (1.433)
	Education	−0.012 (−0.253)	−0.023 (−0.569)	−0.018 (−0.544)
	Marital status	0.084 (0.929)	0.044 (0.561)	−0.019 (−0.297)
Explanatory variables	Perceived usefulness		0.287^**^ (4.184)	0.112 (1.759)
	Perceived of ease of use		0.219^**^ (2.772)	0.172^*^ (2.495)
	Perceived humanity			1.208^**^ (8.100)
	Perceived humanity squared			−0.212^**^ (−8.178)
	Perceived social interactivity			0.176^**^ (2.919)
	Perceived social presence			0.206^**^ (4.174)
Model explanatory degree	*R* ^2^	0.025	0.296	0.525
	Adjusted *R*^2^	0.009	0.277	0.505
	*F* Value	[*F*_(4,235)_ = 1.516, *p* = 0.198]	[*F*_(6,233)_ = 16.292, *p* = 0.000]	[*F*_(10,229)_ = 25.343, *p* = 0.000]
	Δ*R*^2^	0.025	0.27	0.23
	Δ*F* Value	[*F*_(4,235)_ = 1.516, *p* = 0.198]	[*F*_(2,233)_ = 44.716, *p* = 0.000]	[*F*_(4,229)_ = 27.712, *p* = 0.000]

* *p < 0.05*

** *p < 0.01*.

The explanatory variable in this study is Trust, model 1 examines the effect of control variables, and an F-test of the model reveals that the model does not pass the F-test (*F* = 1.516, *p* > 0.05). This indicates that the four control variables of Gender, Age, Education, and Marital Status do not have a significant effect on the logical path of Trust.

The results of model 2 showed that the variation in the F-value was significant (*p* < 0.05) after adding perceived of ease of use and perceived usefulness to model 1. This means that perceived of ease of use and Perceived usefulness added explanatory meaning to the model. In addition, the *R*-squared value increased from 0.025 to 0.296, implying that perceived of ease of use and perceived usefulness can explain 27.0% of the strength of trust. In particular, the regression coefficient value for perceived of ease of use is 0.219 and shows significance (*t* = 2.772, *p* = 0.006 < 0.01), which implies that perceived of ease of use has a significant positive relationship with trust. The regression coefficient value for perceived usefulness is 0.287 and shows significance (*t* = 4.184, *p* = 0.000 < 0.01), which implies that perceived usefulness has a significant positive relationship with Trust.

For model 3, the addition of perceived humanity, perceived humanity squared, perceived social interactivity, and perceived social presence to model 2 produced a significant change in the F-value (*p* < 0.05), implying that the addition of Perceived humanity, perceived humanity squared, perceived social interactivity, and perceived social presence explained the significance of the model. In addition, the R-squared value increased from 0.296 to 0.525, implying that perceived humanity, perceived humanity squared, perceived social interactivity, and perceived social presence can explain 23.0% of the strength of Trust. Specifically, the regression coefficient value for perceived humanity was 1.208 and demonstrated significance (*t* = 8.100, *p* = 0.000 < 0.01), implying that perceived humanity can have a significant positive influence on Trust. The regression coefficient value of perceived humanity squared was −0.212 and showed significance (*t* = −8.178, *p* = 0.000 < 0.01), implying that perceived humanity squared would have a significant negative influence on Trust, which is the inverted U relationship between perceived humanity and Trust. The regression coefficient value of perceived social interactivity is 0.176 and shows significance (*t* = 2.919, *p* = 0.004 < 0.01), implying that perceived social interactivity will have a significant positive influence on Trust. The regression coefficient value of perceived social presence was 0.206 and showed significance (*t* = 4.174, *p* = 0.000 < 0.01), implying that perceived social presence will have a significant positive influence on Trust.

As shown in Table 7, there were 2 models involved in this hierarchical regression analysis. The independent variables in model 1 are control variables (Gender, Age, Education, and Marital Status), and model 2 adds Trust to model 1.

**Table 7:** Multilevel regression test results (Explanatory variable: AVA).

**Category**	**Variable**	**Model 1**	**Model 2**
Control variables	Gender	−0.013 (−0.140)	−0.019 (−0.224)
	Age	0.040 (1.023)	0.015 (0.441)
	Education	0.046 (0.826)	0.048 (0.976)
	Marital status	−0.028 (−0.303)	−0.066 (−0.813)
Explanatory variables	Trust		0.483^**^ (8.074)
Model explanatory degree	Sample size	240	240
	*R* ^2^	0.007	0.224
	Adjusted *R*^2^	−0.01	0.207
	*F* Value	[*F*_(4,235)_ = 0.432, *p* = 0.785]	[*F*_(5,234)_ = 13.477, *p* = 0.000]
	Δ*R*^2^	0.007	0.216
	Δ*F* Value	[*F*_(4,235)_ = 0.432, *p* = 0.785]	[*F*_(1,234)_ = 65.184, *p* = 0.000]

^*^p < 0.05

** *p < 0.01. The t-values are in parentheses*.

The explanatory variable in this study is AVA, model 1 examines the effect of control variables, and an F-test of the model reveals that the model does not pass the F-test (*F* = 0.432, *p* > 0.05). This indicates that the four control variables of Gender, Age, Education, and Marital Status do not have a significant effect on the logical path of AVA. The results of model 2 showed that the variation in the F-value was significant (*p* < 0.05) after adding Trust to model 1. This means Trust added explanatory meaning to the model. In addition, the R-squared value increased from 0.007 to 0.224, implying that Trust can explain 21.6% of the strength of trust. In particular, the regression coefficient value for Trust is 0.483 and shows significance (*t* = 8.074, *p* = 0.000 < 0.01), which implies that Trust has a significant positive relationship with AVA.

### Intermediation Effect Test

In this paper, the bias-corrected nonparametric percentile bootstrap method was applied to test the mediating effect (as shown in Table 8), and the confidence level was set at 95%.

**Table 8:** Summary of intermediary role test results.

**Items**	**Total effect**	**Intermediary effect**	**95% BootCI**	**Direct effect**	**Effectiveness ratio**	**Test conclusion**
Functionality-trust-AVA	0.584^**^	0.058	0.009~0.107	0.525^**^ (0.400~0.655)	10.017%	Partial mediation
Social emotion-trust-AVA	0.079	0.058	0.011~0.127	0.017 (−0.091~0.136)	100%	complete mediation

^*^p < 0.05,

** *p < 0.01*.

In the path from functionality to acceptance, the 95% BootCI was (0.012~0.109), excluding zero; the trust interval for direct effects was (0.398~0.652), excluding zero, indicating that it was partially mediated. In the path from social emotion to acceptance, the 95% BootCI was (0.013~0.129), excluding zero; the direct effect trust interval was (−0.096~0.130), including zero, indicating that trust plays a full mediating effect in the process of moving from social emotion to acceptance, and hypotheses 4 and 5 are confirmed.

**Figure 2:**
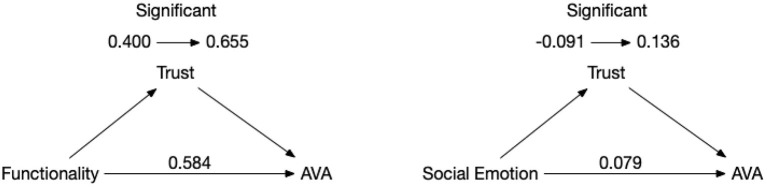
Intermediary role test results.

The intermediary role test results were shown in Figure 2.

## Discussion

This paper develops an AI virtual assistant acceptance model based on the technology acceptance model and the service robot acceptance model. Overall, the AVA model extends the potential path of AVA and improves the study of the acceptance transformation mechanism from the trust perspective. The model shows high predictive power for AVA and can well explain the differences in user acceptance and the reasons for the formation of differences. The results of the study can be summarized in three aspects as follows.

First, this paper verifies the positive effect of trust on the acceptance of AI virtual assistants. The services of AI virtual assistants are executed based on AI algorithms, but users do not fully trust the information or services provided by AI virtual assistants due to the inherent black-box problem of AI technology. Existing studies suggest that trust is an essential driver of technology acceptance and can positively influence users' acceptance of new technologies (Kaplan and Haenlein, 2019; van Pinxteren et al., 2019; Asatiani et al., 2020). This paper confirms through empirical research that trust is significantly and positively correlated with AI virtual assistant acceptance, and that its ability to reduce users' negative emotions toward AI virtual assistants plays a key role in improving AI virtual assistant acceptance.

Second, this study explored the relationship with trust at the functionality and social emotional levels. Most of the existing studies focus on the relationship between functionality and acceptance (King and He, 2006), but there is a lack of research on the relationship with trust. Based on the technology acceptance model, this paper examines the effect of functionality on trust in terms of two dimensions, perceived usefulness and perceived of ease of use, which confirmed that both usefulness and ease of use dimensions were significantly and positively related to trust. This shows that efficient service experience helps users develop trust, which means that effective services of AI virtual assistants will encourage users to increase their interactions with them and thus develop trust based on familiarity with their functions.

In addition, the degree of user trust in AI virtual assistants depends on their ability to satisfy users' social-emotional and relational needs. Drawing on the service robot acceptance model, this paper divides social emotion into three dimensions: perceived humanity, perceived social interactivity, and perceived social presence. Currently, there are two different views on perceived humanity. Social reaction theory and social presence theory believe that higher anthropomorphism will lead to a positive customer response, which means that perceived humanity will lead to user trust (Qiu and Benbasat, 2009; van Doorn et al., 2017). Conversely, some scholars have argued that the positive effects of highly anthropomorphic AI virtual assistants fail to be proven in many scenarios and can even increase users' negative emotions (Tinwell et al., 2011; van Doorn et al., 2017). Consequently, this paper explores the relationship between perceived humanity and trust through empirical tests and finds a non-linear inverted U-shaped relationship between perceived humanity and trust, meaning that only moderate perceived humanity can promote trust among users. In addition, the maturation of AI technology will enable AI virtual assistants to have certain human-like attributes, such as voice, real-time interaction, verbal communication skills, and social etiquette, that enable human users to perceive their social presence. Scholars believe that these attributes can generate positive emotions and establish favorable social relationships, which can enhance the interaction between users and AI virtual assistants to help increase their trust level (Fernandes and Oliveira, 2021). This study further confirmed that perceived social interactivity and perceived social presence were positively related to trusting behavior, which is consistent with the findings of existing studies.

Finally, this paper explores the mediating role of trust between functionality and social emotion and acceptance. Trust motivates AI virtual assistants to build a favorable image and suppresses users' perceptions of various risks, which in turn positively motivates users' acceptance behavior toward them. According to Schmitt (1999), customers' purchasing behavior is the result of a combination of rational and emotional factors. Drawing on the customer delivered value theory, AI virtual assistants should make every effort to provide customers with quality services, obtain customer satisfaction, and help customers generate willingness to use. AI virtual assistants that provide favorable services give users a comfortable experience, which generates a brand effect, increases user loyalty and trust, and forms a positive cycle. This paper confirms the mediating role of trust between functionality and acceptance, with the service experience being the bridge between users and AI virtual assistants to build a well-trusted relationship. The social emotions of AI virtual assistants enable users to create sublimation and association with social systems, which leads to satisfaction at the psychological level. Trust is the prerequisite for user identification and the key factor that determines whether users are willing to interact deeply with the information source (Wirtz et al., 2018). Based on the above studies, this paper confirms the mediating role of trust between social emotion and acceptance, which is important for enhancing the contextualized services of AI virtual assistants from a perceptual perspective.

In summary, this study establishes a new acceptance model for AI virtual assistants, verifying the inverted U-shaped effect of perceived humanity on trust and the mediating role of trust in the acceptance transformation mechanism. It complements the gap of existing technology acceptance models at the trust level and expands the transformation path of AVA. To a certain extent, it extends the boundary and application space of existing theories and helps to solve the user acceptance problem from the trust perspective.

## Data Availability Statement

The raw data supporting the conclusions of this article will be made available by the authors, without undue reservation.

## Ethics Statement

Ethical review and approval was not required for the study on human participants in accordance with the local legislation and institutional requirements. The patients/participants provided their written informed consent to participate in this study. Written informed consent was obtained from the individual(s) for the publication of any potentially identifiable images or data included in this article.

## Author Contributions

SZ and ZM: writing. BC and XY: providing revised advice. XZ processing data. All authors contributed to the article and approved the submitted version.

## Conflict of Interest

The authors declare that the research was conducted in the absence of any commercial or financial relationships that could be construed as a potential conflict of interest.

## Publisher's Note

All claims expressed in this article are solely those of the authors and do not necessarily represent those of their affiliated organizations, or those of the publisher, the editors and the reviewers. Any product that may be evaluated in this article, or claim that may be made by its manufacturer, is not guaranteed or endorsed by the publisher.
