# Erratum: Motivation, Social Emotion, and the Acceptance of Artificial Intelligence Virtual Assistants—Trust-Based Mediating Effects

**DOI:** 10.3389/fpsyg.2021.790448

**Published:** 2021-10-26

**Authors:** Frontiers Production Office

**Affiliations:** Frontiers Media SA, Lausanne, Switzerland

**Keywords:** AI virtual assistant, motivation, social emotion, trust, acceptance, mediating effects, inverted U relationship

Due to a production error, an incorrect funding statement was inserted into the article.

A correction has been made to the **Funding** section.

“This work was supported by the Social Science Foundation of Heilongjiang Province of China (18TQC239), Guangxi Science and Technology Plan Project (Guangxi Science and Technology Base and Talent Special Project: AD20159069).”

The publisher apologizes for this mistake. The original version of this article has been updated.

## Publisher's Note

All claims expressed in this article are solely those of the authors and do not necessarily represent those of their affiliated organizations, or those of the publisher, the editors and the reviewers. Any product that may be evaluated in this article, or claim that may be made by its manufacturer, is not guaranteed or endorsed by the publisher.

